# Effects of dissolved organic phase composition and salinity on the engineered sulfate application in a flow-through system

**DOI:** 10.1007/s11356-020-07696-6

**Published:** 2020-01-24

**Authors:** Saeid Shafieiyoun, Riyadh I. Al-Raoush, Reem Elfatih Ismail, Stephane K. Ngueleu, Fereidoun Rezanezhad, Philippe Van Cappellen

**Affiliations:** 1 Department of Civil and Architectural Engineering, College of Engineering, Qatar University, PO Box 2713, Doha, Qatar; 2 Ecohydrology Research Group and Water Institute, Department of Earth and Environmental Sciences, University of Waterloo, 200 University Avenue West, Waterloo, Ontario N2L 3G1 Canada

**Keywords:** Multi-component NAPL, Dissolved organic phase, Sulfate injection, Salinity, Hydrocarbon-contaminated groundwater, Sulfate reduction

## Abstract

Engineered sulfate application has been proposed as an effective remedy to enhance the rate-limited biodegradation of petroleum-hydrocarbon-contaminated subsurface environments, but the effects of dissolved organic phase composition and salinity on the efficiency of this method are unknown. A series of flow-through experiments were conducted for 150 days and dissolved benzene, toluene, naphthalene, and 1-methylnaphthalene were injected under sulfate-reducing and three different salinity conditions for 80 pore volumes. Then, polycyclic aromatic hydrocarbons (PAHs) were omitted from the influent solution and just dissolved benzene and toluene were injected to investigate the influence of dissolved phase composition on treatment efficiency. A stronger sorption capacity for PAHs was observed and the retardation of the injected organic compounds followed the order of benzene < toluene < naphthalene < 1-methylnaphthalene. Mass balance analyses indicated that 50 and 15% of toluene and 1-methlynaphtalene were degraded, respectively. Around 5% of the injected naphthalene degraded after injecting > 60 PVs influent solution, and benzene slightly degraded following the removal of PAH compounds. The results showed substrate interactions and composition can result in rate-limited and insufficient biodegradation. Similar reducing conditions and organic utilization were observed for different salinity conditions in the presence of the multi-component dissolved organic phase. This was attributed to the dominant microbial community involved in toluene degradation that exerted catabolic repression on the simultaneous utilization of other organic compounds and were not susceptible to changes in salinity.

**Electronic supplementary material:**

The online version of this article (10.1007/s11356-020-07696-6) contains supplementary material, which is available to authorized users.

## Introduction

Soil and groundwater contamination resulting from the release of petroleum hydrocarbons can pose significant risks and carcinogenic effects into the human health. Following release near the ground surface, organic hydrocarbons start a complex migration and distribute as a non-aqueous phase liquid (NAPL) in the subsurface system (Gan et al. 2009; Malk et al. 2014; Al-Raoush 2014; Acosta-gonzález et al. 2015). Anaerobic biodegradation is the most dominant process in the organic-contaminated soil and groundwater due to depletion of dissolved oxygen by aerobic respiration processes (Anderson and Lovley 1997; Perelo 2010). Sulfate is one of the most important electron acceptors in subsurface systems that can be reduced by sulfate-reducing bacteria (SRB) and contributes ~ 70% of the natural anaerobic biodegradation (Miao et al. 2012; Suthersan et al. 2011). Due to depletion of terminal electron acceptors during anaerobic biodegradation processes in the contaminated soil and groundwater, biodegradation of organic contaminates will be restricted resulting in a long remediation timeframe (Meckenstock et al. 2015). Hence, supplying sulfate as a remediation strategy to enhance the indigenous SRB community and biodegradation process has been proposed in the recent years (Kauppi et al. 2011; Simpanen et al. 2016; Müller et al. 2017; Wei et al. 2018).

Although published studies about the bioremediation of organic-contaminated subsurface systems have been divers in terms of experimental methodology, they indicate that substrate interactions and dissolved organic phase composition can significantly affect the capabilities of microbial communities and hence, influence the thermodynamics and kinetic aspects of the biodegradation process (Meckenstock et al. 2015; Fowler et al. 2016). For example, previous studies indicate that during anaerobic degradation of benzene, toluene, xylene, and ethylbenzene (BTEX) under sulfate-reducing conditions, initially toluene and xylene will be degraded and then benzene and ethylbenzene might be oxidized (Cunningham et al. 2001; Edwards and Garbic-Galic 1992). Edwards et al. (1992) reported that while toluene, p-xylene, and o-xylene were degraded under sulfate-reducing condition, benzene and ethylbenzene were not degraded. Edwards and Garbic-Galic (1992) in another microcosm study, where the sediment was just amended with benzene, showed the potential anaerobic degradation of benzene under sulfate-reducing condition after a 30-days lag time. However, benzene degradation rate was slower once the microcosms were amended by both toluene and benzene. Meckenstock et al. (2004) also observed that o-xylene degradation was inhibited by toluene and once toluene was omitted from the system, o-xylene was utilized. They isolated two different types of SRB species from their soil samples and found that while one of the strains could degrade both toluene and o-xylene as the sole substrate, presence of toluene could inhibit o-xylene degradation. Similar to the experimental systems, field-scale applications of sulfate for the remediation of the BTEX-contaminated groundwater manifest that dissolved organic phase composition can control biodegradation effectiveness. Kolhatkar and Schnobrich (2017) monitored a benzene-dominated plume over 4 years and reported that dissolved benzene concentration declined following the land application of sulfate salt. However, Davis et al. (1999) identified no evidence of benzene degradation for a BTEX plume in a sulfate-rich groundwater during a 5-year field study. Cunningham et al. (2001) also reported that benzene can be anaerobically degraded under simultaneous injection of sulfate and nitrate after other preferentially biodegraded organic compounds were removed.

Polycyclic aromatic hydrocarbons (PAHs) (i.e., naphthalene, methylnaphthalene) are another organic compound class which are abundant in the hydrocarbon-contaminated subsurface systems. Anaerobic degradation of PAHs has been also reported specifically when they are present as the sole carbon and energy source (Galushko et al. 1999; Musat et al. 2009; Meckenstock et al. 2016; Ngueleu et al. (2019)). Galushko et al. (1999) demonstrated that naphthalene was completely oxidized during a series of SRB enrichment experiments by using marine sediments. Musat et al. (2009) also observed anaerobic degradation of naphthalene by SRB originated from a Mediterranean sediment. The naphthalene-grown strains were then exposed to 2-mtylnaphthalene to investigate their adaptation capability. The results showed that 2-methylnaphthalne was utilized after more than 20 days of lag time indicating that the capacity for 2-methylnaphthalene degradation was not induced during the naphthalene degradation process. Thierrin et al. (1993) performed a series of groundwater test and mathematical modeling to estimate degradation rates of the dissolved BTEX and naphthalene under sulfate-reducing conditions. They concluded that toluene and naphthalene had the highest (14.1 kg year^−1^) and lowest (0.41 kg year^−1^) degradation rates, respectively, and benzene was not degraded. Wei et al. (2018) executed a pilot-scale experiment to investigate episodical surface-based sulfate application for the remediation of a petroleum-contaminated source zone. Despite some fluctuation in dissolved BTX concentrations, no significant change was observed for the dissolved naphthalene concentration. However, they reported that the dissolved organic concentrations might be affected by changes in groundwater flow regime caused by sulfate application.

It can be concluded that while many of the common petroleum hydrocarbons typically found in the contaminated subsurface systems can be anaerobically biodegraded especially when they are the sole organic compound and energy source (Meckenstock et al. 2016; Lee et al. 2019), the behavior of the multi-component NAPLs is uncertain due to the presence of more available substrates, catabolite repression, and competitive biodegradation (Meckenstock et al. 2015). However, quantifying and predicting the influence of substrate interactions and multi-component dissolved organic phase resulted from different NAPL compositions on biodegradation process are crucial to develop effective remediation strategies. Although a few studies have investigated the effects of substrate interactions on hydrocarbon degradation under sulfate-reducing conditions (Bally and Egli 1996; Lovanh and Alvarez 2004), most of these studies focused on different genetic aspects or enzyme activities. However, design and implementation of remediation strategies for different NAPL compositions require a better understanding of the indigenous microbial community responses under realistic dynamic subsurface conditions.

In addition to NAPL composition and substrate interactions which can result in rate-limited and insufficient biodegradation, biogeochemical and environmental conditions of the subsurface systems can also affect the growth of microbial community (Meckenstock et al. 2015) and hence treatment effectiveness. For example, salinity in the subsurface systems can affect the biodegradation processes. Rhykerd et al. (1995) performed a series of aerobic slurry batch experiments and reported an inverse dependency between the oil degradation efficiency and electrical conductivity (EC). However, to the best of our knowledge, there is no study that investigates the effects of salinity on the petroleum-contaminated subsurface responses to the sulfate application. Additionally, the effect of salinity on the substrate interactions is also unknown.

The main objective of this study was to explore the simultaneous effects of dissolved organic phase composition and salinity on the efficiency of the engineered sulfate application in the anaerobic petroleum-contaminated subsurface systems. A series of flow-through experiments were constructed using undisturbed soil samples collected from a (semi)-arid coastal region to simulate dynamic anaerobic-contaminated subsurface conditions. Flow-through reactors (FTRs) were injected by a synthetic sulfate solution containing dissolved benzene, toluene, naphthalene, and 1-methylnaphthalene as samples of BTEX and PAH compounds and with three different salinity conditions. The relevant geochemical indicators were monitored and after development of reducing condition and achieving steady conditions, PAH compounds were omitted from the injected solution to investigate adaptation capability and compare the biodegradation of two different dissolved organic phases. Specifically, the focus was to compare the response of contaminated subsurface systems under sulfate-reducing conditions between two different organic compound classes (BTEX and PAHs).

## Materials and methods

### Field site and soil sampling

Soil samples were collected from a coastal region known as Sumaysimah beach on the eastern coast of the state of Qatar (25°34′26.61″N; 51°29′18.77″E). To ensure the geochemical and microbial properties were not affected, non-contaminated undisturbed soil samples were collected from the depths of 0 to 10 cm above the groundwater table that gently and quickly capped. The soil texture was silty-sandy and organic matter content was negligible and mostly related to small fraction of plant residues with dimeters < 4 mm. The particle density of the soil was 2.72 kg L^−1^ and mechanical sieving analyses indicated that specific particle diameters equivalent to 10 and 60% passing (d_10_ and d_60_) are 0.11 and 0.58 mm, respectively (Ngueleu et al. 2018). Although mineralogical analyses of the soil samples were not performed, calcite is known to be the main mineral composition in this soil (Shomar 2015; Yigiterhan et al. 2018; Ngueleu et al. 2018). Pore water pH and EC at the sampling location were measured as ~ 7.5 and ~ 4000 μS cm^−1^, respectively, which can be categorized in the range of brackish waters.

### Flow-through reactor instrumentation

**Fig. 1:**
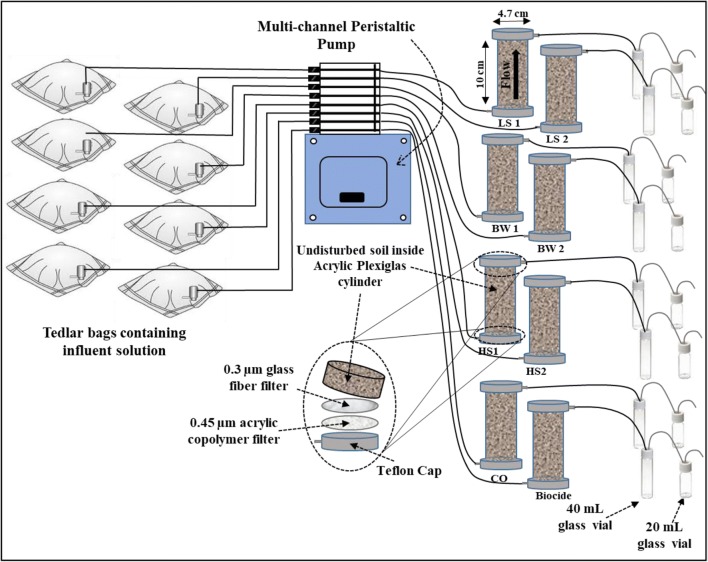
Schematic diagram of the flow-through reactor (FTR) setup to investigate biodegradation of multi-component dissolved phase organic compounds under sulfate-reducing condition for the low-salinity (LS), brackish water (BW), and high-salinity (HS) conditions

A total of eight flow-through reactors (FTRs) were operated for a 150-day experimental period. Each FTR was constructed similar to Pallud et al. (2007) by using a Plexiglas tube (10 cm length and 4.7 cm ID) which is used to collect the undisturbed soil. The Plexiglas tubes were attached to polyvinyl chloride (PVC) endcaps with an opening for tubing connection. Between each endcap and the soil sample, an O-ring, a 0.45-μm pore size acrylic copolymer disk filter (VWR International), and a 1-μm pore size glass fiber filter (VWR International) were placed to act as a flow distributer. The FTRs were connected to a peristaltic pump (Masterflex) from the bottom endcap and the top endcap had an outflow channel to conduct the effluent into sampling vials. PTFE Teflon tubes (1/8″ O.D. × 0.063″ I.D., Sigma-Aldrich) were used for inflow and outflow channels and a flow rate of 2.7 mL h^−1^ was used during the entire experiment, to be in the range of groundwater flow rate at the sampling location. FTRs were covered by aluminum foil to simulate subsurface dark conditions. A schematic diagram of the column setup is shown in Fig. 1 (A picture of the experimental setup is provided as Supplementary Material (SM), Figure SM1.). This system was designed to simulate realistic dynamic and anaerobic subsurface conditions and also to minimize evaporation of dissolved organic phase during effluent sampling.

### Synthetic influent solution

The FTRs were injected by a synthetic influent solution prepared based on groundwater sample analysis from the sampling location containing background nutrients including MgCl_2_ (89 mg L^−1^), KCl (24 mg L^−1^), and CaCl_2_.H_2_O (243 mg L^−1^) in argon-purged deoxygenated Milli-Q water (DO < 0.8 mg L^−1^) to provide anaerobic conditions in the FTRs. Sulfate concentration in the synthetic influent solution was 150 mg L^−1^ to be in the range of practical applicable concentrations for SRB in the experimental systems due to formation of inhibitory sulfide (Cunningham et al. 2001). At the beginning of the experiments, an organic mixture composed of four organic compounds including benzene (3.5 mg L^−1^), toluene (3.5 mg L^−1^), naphthalene (1.5 mg L^−1^), and 1-methylnaphthalene (1.5 mg L^−1^) as samples of BTEX and PAH compounds was dissolved in the synthetic influent solution. After ~ 80 PVs once the steady condition was achieved, PAH compounds were omitted and just benzene and toluene with the initial concentration of 5 mg L^−1^ were added to the influent solution to explore the effects of dissolved organic phase composition on subsurface responses to the engineered sulfate injection. Based on a previous adsorption experiments on the same soil samples (Ngueleu et al. 2018), 150 mg L^−1^ sulfate concentration in the synthetic influent solution was more than the stoichiometric requirement for the reaction with all organic compounds during the experimental period. This high sulfate concentration was supplied to ensure SRB development over potentially competing microbes. The synthetic influent solution pH was adjusted to be ~ 7 using HCl and NaOH and then transferred into separate 1-L Tedlar bags (Sigma-Aldrich) which served as reservoirs for the FTRs using a peristaltic pump under anaerobic conditions in a glove bag filled by argon gas (Figure SM1). Tedlar bags were employed to secure the influent solution against oxygen penetration.

### FTR experimental approach

Two FTRs were injected by the synthetic influent solution containing background nutrients, sulfate, and the organic compounds that had low salinity (EC = ~ 1000 μS cm^−1^) representing fresh water conditions and identified as LS1 and LS2. To investigate the effects of brackish environments similar to the sampling location, two FTRs were injected by the synthetic influent solution prepared similar to the LS FTRs but 1300 mg L^−1^ of NaCl was added to increase the EC to the brackish water level (~ 4000 μS cm^−1^) at the sampling location (identified as BW1 and BW2). To investigate the effects of high-salinity environments, two FTRs were injected by the synthetic influent solution prepared similar to the LS FTRs but 20,000 mg L^−1^ of NaCl was added to increase the EC to the seawater level (25,000 μS cm^−1^) (identified as HS1 and HS2). To identify the naphthalene adsorption to the soil, one FTR (identified as Biocide) was also injected identical to the LS FTRs but 275.5 mg L^−1^ of HgCl_2_ biocide was added to the synthetic influent solution to inhibit biodegradation (Van De Graaf et al. 1995). The last FTR was injected by a synthetic solution containing just background nutrients and the organic compounds (no sulfate) and identified as CO. It was intended to compare the effects of sulfate addition with organic removal due to natural attenuation and under potential methanogenic conditions.

The following sequence of steps were applied during the 150-day experimental period at room temperature (23 ± 2 °C): (1) 3 pore volumes (PVs) of Milli-Q water containing background nutrients were injected to displace pore water; (2) 3 PVs of argon-purged 100 mg L^−1^ of NaBr solution were injected to investigate hydraulic consistency through the analysis of bromide (Br^−^) breakthrough curve; (3) FTRs were injected for 30 days by argon-purged synthetic influent solution containing background nutrients (no sulfate and organic compounds) to provide anaerobic saturation conditions; (4) FTRs were injected for 80 days (~ 80 PVs) by argon-purged synthetic influent solution containing background nutrients, sulfate, and four organic compounds (3.5 mg L^−1^ benzene, 3.5 mg L^−1^ toluene, 1.5 mg L^−1^ naphthalene, 1.5 mg L^−1^ 1-methylnaphthalene); and (5) naphthalene and 1-methylnaphthalene were omitted from the influent solution and concentrations of benzene and toluene were increased to 5 mg L^−1^ and then FTRs were injected again for 40 days (~ 30 PVs).

### FTR effluent sampling

The influent and effluent solutions were sampled regularly and analyzed for the concentrations of organic compounds, sulfate, and dissolved inorganic carbon (DIC). In addition, pH, EC, dissolved oxygen (DO), and redox potential (Eh) of the effluent samples were also measured. To minimize organic compound volatilization in the effluent samples, the outlet of the FTRs was connected to 40-mL and 20-mL glass vials in series. Both vials were always kept full with the aqueous phase and effluent samples were collected from the 40-mL vial which was not in contact with air (Fig. 1).

### Preliminary experiments with single dissolved organic compound

A series of preliminary FTRs were also executed (data not provided here) and naphthalene or lactate as a single carbon source (instead of the four organic compounds used in this study) was injected separately under the experimental conditions similar to the LS and BW FTRs to identify SRB activities. Lactate is a preferred carbon source for bacteria and provides the most ideal conditions for SRB development. The preliminary experiments with dissolved lactate confirmed the development of reducing conditions, sulfate consumption, and SRB activities under the experimental conditions. The results from the preliminary single carbon source FTRs were attributed to this study and while methanogenesis process could also occur within the FTRs, we assumed that the SRB will outperform methanogens due to long-term continuous supply of sulfate (Dar et al. 2008; Stams et al. 2005; Talbot et al. 2008). Although a series of culture-based or genetic-based microbial analyses could provide additional lines of evidence to better identify the main electron acceptors, for the purpose of this study, the combination of geochemical indicators such as redox potential, DO, and DIC concentrations along with sulfate consumption during 120 days of sulfate injection was deemed sufficient to investigate hydrocarbon-contaminated subsurface responses to the engineered sulfate injection and separate microbial analyses were not in the scope of this study.

### Reagents and analytical methods

Naphthalene (C_10_H_8_, Sigma-Aldrich), benzene (C_6_H_6_, Sigma-Aldrich), toluene (C_7_H_8_, Sigma-Aldrich), 1-methylnaphthalene (C_11_H_10_, Sigma-Aldrich), sodium sulfate (Na_2_SO_4_, Sigma-Aldrich), mercuric chloride (HgCl_2_, Sigma-Aldrich), manganese chloride (MgCl_2_, Sigma-Aldrich), potassium chloride (KCl, Sigma-Aldrich), calcium chloride (CaCl_2_.H_2_O, Sigma-Aldrich), sodium chloride (NaCl, Sigma-Aldrich), sodium bromide (NaBr, Sigma-Aldrich), and methylene chloride (CH_2_Cl_2_, EMD Millipore) were all of reagent grade and used as received.

For organic analyses of the aqueous samples, a gas chromatography (Clarus 680, Perkin Elmer) with flame ionization detector (FID) connected to a head space auto sampler (Turbo Matrix 40 trap, Perkin Elmer) was used. Temperature of headspace’s vial, needle, and transfer line was 85 °C, 90 °C, and 110 °C, respectively. Vials were shaken for 15 min in the headspace oven at 85 °C. Samples were injected onto a 0.25 mm × 60 m length, PerkinElmer Elite-1 capillary column with a stationary phase film thickness of 0.25 μm. Helium column flow rate was 1 mL min^−1^ and the FID detector temperature was 250 °C. The oven program started from 40 °C as an initial temperature held for 18 min, then ramped at 7.0 °C min^−1^ to a final temperature of 250 °C and held for 10 min. Chromatographic run time for each sample was 58 min.

Aqueous effluents were analyzed for ion chemistry using an 850 Professional IC (Metrohm) ion chromatograph equipped with two conductivity detectors (anion and cation) to measure the concentrations of Br^−^, sulfate, and nitrate during the tracer tests. Injected volume of sample was 20 μL for anions of interest and the column used was “Metrosep A Supp 5 - 150/4.0.” The mobile phase was carbonate eluent: sodium hydrogen carbonate 1.0 mmol/L, sodium carbonate 3.2 mmol/L, ultrapure water as rinsing solution, and 100 mM H_2_SO_4_ as regeneration solution. Data from ion chromatography were processed using “MagIC Net” software.

An Orion™ Versa Star Pro™ Benchtop Meter (Thermo Scientific) was used to measure pH, EC, Eh, and DO in the aqueous effluent samples. Eh was measured immediately after removing the cap of the 40-mL sampling vial and then pH and EC were measured. Around 20 mL of sample was collected for GC-FID analyses. At the end of sampling process, effluent samples for DIC, and sulfate concentration were collected. Sulfate concentration was measured based on APHA 4500-SO4.E method (Rice et al. 2017) using a HACH (DR5600) laboratory spectrophotometer. DIC concentrations of effluent samples were determined using a total organic carbon analyzer (SKALAR, Formacs^HT/TN^ TOC/TN Analyzer). Each sample was analyzed three times and average values were reported along with associated standard deviations.

## Results and discussions

The effective porosity in each FTR was calculated using the tracer breakthrough curve analyses of the measured Br^−^ concentrations (Figure SM2) (Weber and DiGiano 1995). The effective porosity for all the FTRs was in the range of 40 to 45%. Table 1 provides a summary of the effective porosities and operational conditions for the FTRs. Because undisturbed soil samples were used for the experiments, the effective porosity and the total injected PVs were different for each FTR.

**Table 1:** Summary of the flow-through reactor (FTR) specifications and operational conditions

FTRs	Effective		
	Porosity	Total injection	Synthetic influent solution^1^
	(%)	(PV)	
LS1	42	102	Nutrients^2^, sulfate^3^, organic compounds^4^
LS2	45	94	Nutrients^2^, sulfate^3^, organic compounds^4^
BW1	44	98	Nutrients^2^, sulfate^3^, organic compounds^4^, NaCl^5^
BW2	44	98	Nutrients^2^, sulfate^3^, organic compounds^4^, NaCl^5^
HS1	42	102	Nutrients^2^, sulfate^3^, organic compounds^4^, NaCl^6^
HS2	42	102	Nutrients^2^, sulfate^3^, organic compounds^4^, NaCl^6^
Biocide	43	100	Nutrients^2^, sulfate^3^, organic compounds^4^ HgCl_2_^7^
CO	40	108	Nutrients^2^, organic compounds^4^

^1^All the chemicals were dissolved in argon-purged Milli-Q water (DO < 0.8 mg L^−1^)

^2^Nutrients include MgCl_2_ (89 mg L^−1^), KCl (24 mg L^−1^), and CaCl_2_.H_2_O (243 mg L^−1^)

^3^Sulfate concentration was 150 mg L^−1^

^4^Organic compounds include benzene, toluene, naphthalene, and 1-methylnaphthalene

^5^NaCl concentration in the brackish water (BW) FTRs was 1300 mg L^−1^

^6^NaCl concentration in the high-salinity (HS) FTRs was 20,000 mg L^−1^

^7^HgCl_2_ concentration was 275.5 mg L^−1^

The effluent EC values during the experimental period were not affected compared to those from the influent solutions and were in the range of 1000, 4000, and 25,000 μS cm^−1^ for the LS, BW, and HS FTRs, respectively. Prior to the injection of dissolved sulfate and organic compounds, FTRs were injected with synthetic solution containing background nutrients for 30 days and hence, the concentrations of dissolved sulfate and nitrate from the soil content reached less than the method detection limit (MDL). The effluent pH for all FTRs increased from 7 (in the influent solution) to the range of 8–9 probably due to the soil alkalinity and production of weaker acids during sulfate reduction. Slight black color was observed at the outlet of the BW and HS FTRs after 60 days of sulfate injection which can be attributed to the precipitation of iron sulfide produced during the reaction of iron salts from soil content and generated hydrogen sulfide due to sulfate reduction (Acton and Barker 1992). Because FTRs were injected by the synthetic solution prior to the sulfate injection, a significant amount of iron from the soil content was washed out and hence, no significant iron sulfide precipitation was expected due to SRB activities.

### Development of reducing conditions

**Fig. 2:**
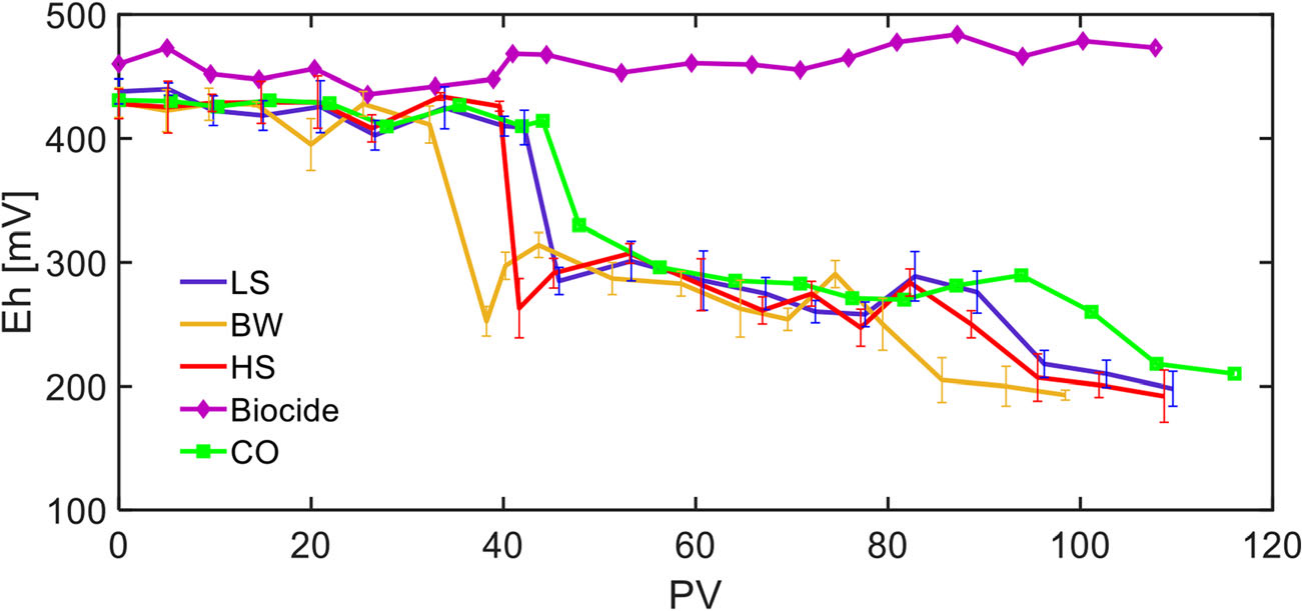
Effluent Eh values from the flow-through reactors (FTRs). Due to differences in the effective porosity of the undisturbed soil sample, the numbers of injected PVs in each FTR are not the same. Each data point for the low-salinity (LS), brackish water (BW), and high-salinity (HS) FTRs represents the average of single measurements from the duplicate reactors and the error bars represent the range of data. Each data point for the Biocide and control (CO) FTRs is related to single measurement from one reactor

Effluent Eh values from the FTRs are provided in Fig. 2. For the Biocide FTR, effluent Eh values did not change during the experimental period and fluctuated between + 450 and + 500 mV which was similar to the Eh values of the synthetic influent solution and indicates that microbial activities were successfully limited and reducing condition was not developed. After 40 days of lag time and injecting > 30 PVs of the influent solution, effluent Eh values dropped to < + 300 mV initially in the BW and HS FTRs, followed by the LS FTRs and finally by the CO FTR. After injecting 80 PVs and omitting naphthalene and 1-methylanphthalene from the influent solution, effluent Eh values of all the FTRs except Biocide again dropped and reached to ~ + 200 mV, suggesting that PAHs affected the benzene and toluene degradation process similar to the biocide substance and increased the effluent Eh. The effluent Eh from the CO FTR dropped after that of the LS, BW, and HS FTRs and can be attributed to the development of methanogens which can dominate in the absence of other electron acceptors (Dar et al. 2008).

Overall, the high effluent Eh values from the Biocide FTR during the experimental period established a baseline signature and significant effluent Eh reduction in the LS, BW, HS, and CO FTRs confirmed the development of reducing conditions and microbial activities under the experimental conditions. While redox potentials for different salinity conditions were similar in the presence of the multi-component dissolved organic phase and no significant difference was observed between Eh values from the LS, BW, HS, and CO FTRs, after omitting naphthalene and 1-methylnaphthalen (80 PVs) from the influent solution, Eh values decreased indicating that reducing conditions and microbial activities were enhanced. It can be concluded that dissolved phase composition can influence microbial activities and treatment efficiencies.

The results from the preliminary FTR experiments injected with lactate or 4 mg L^−1^ dissolved naphthalene indicated that the carbon source can significantly affect the redox condition and microbial activities and subsequently biodegradation process (data not shown here). Effluent Eh values from the preliminary FTRs injected by lactate reached to < − 50 mV which is the most ideal conditions for SRB activities (Cassidy et al. 2015). In addition, effluent Eh values during the preliminary FTR experiments injected by 4 mg L^−1^ dissolved naphthalene, as a single carbon source, were different for the LS and BW FTRs and in the range of + 200 and + 300 mV, respectively.

Two differences were observed between the effluent Eh values from the FTRs injected with multi-component dissolved organic phase in this study and those from preliminary FTRs injected with dissolved naphthalene as a single carbon source. While the results of this study indicate salinity has no significant influence on effluent Eh values in the presence of multi-component dissolved organic phase, the results from the preliminary FTRs injected by dissolved naphthalene indicated effluent Eh values were significantly different for the LS and BW conditions. In addition, while the injection of dissolved naphthalene resulted in the effluent Eh value of + 200 mV for the BW conditions, presence of naphthalene and 1-methylnaphthlene along with benzene and toluene resulted in the Eh value of + 250 to + 300 mV and after omitting the PAHs dropped to 200 mV. It can be concluded that development of reducing conditions and microbial activities are inhibited in the presence of complex dissolved organic phase. However, evaluation of effluent organic concentrations is required to understand why the effluent Eh values under different salinity conditions were similar in the presence of multi-component dissolved organic phase in this study as opposed to those for the single dissolved organic compound (see the “Effluent organic concentrations” section).

### Effluent organic concentrations

**Fig. 3:**
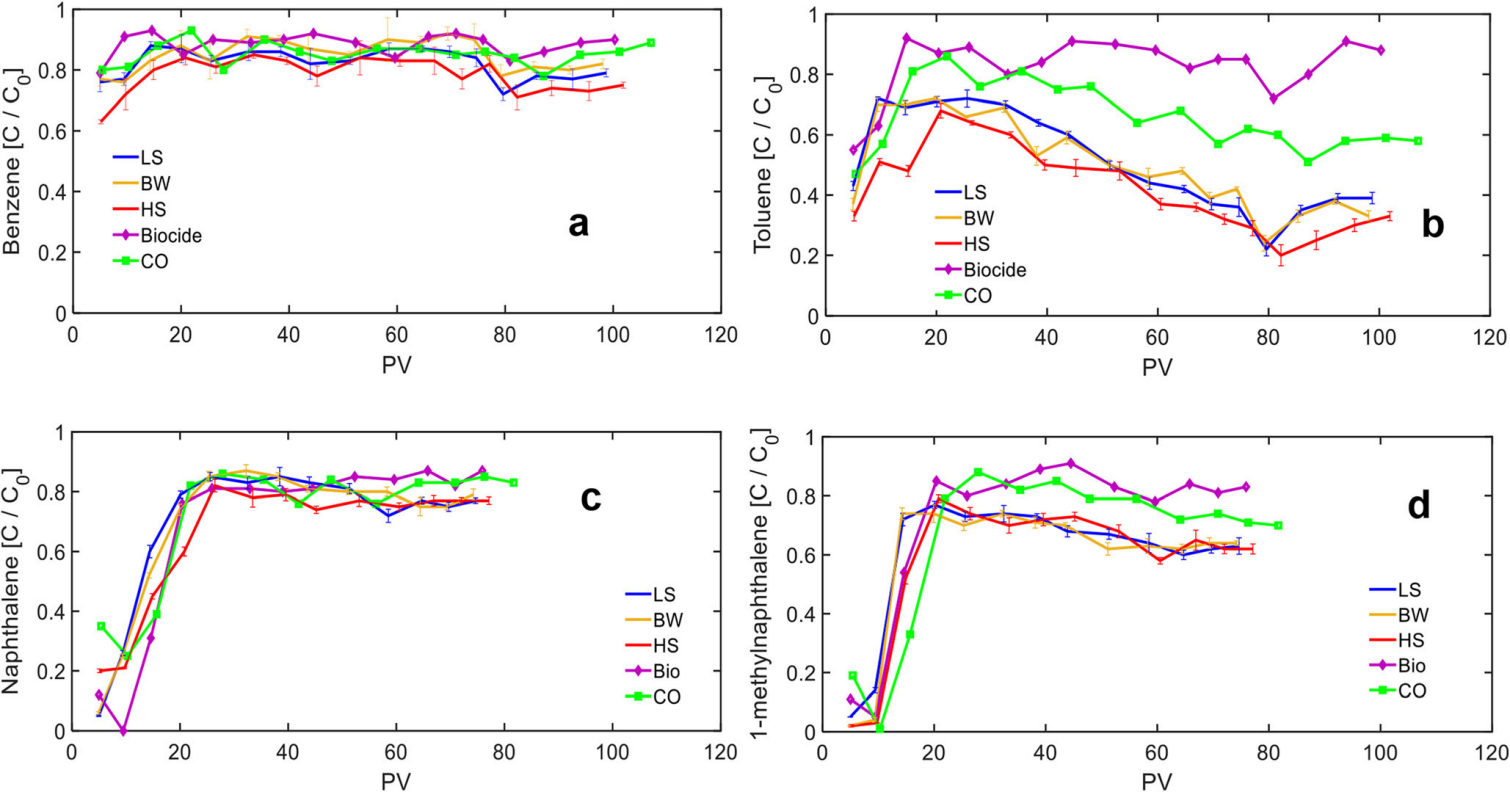
Ratio of effluent concentrations to initial injected concentrations of a) benzene, b) toluene, c) naphthalene, and d)1-methylnaphthalene from all flow-through reactors (FTRs). Each data point for the low-salinity (LS), brackish water (BW), and high-salinity (HS) FTRs represents the average of single measurements from the duplicate reactors and the error bars represent the range of data. Each data point for the Biocide and control (CO) FTRs is related to single measurement from one reactor. Naphthalene and 1-methylnaphthalene were injected for 80 PVs and then omitted from the influent solution but benzene and toluene were injected for ~ 110 PVs. Due to differences in the effective porosity of the undisturbed soil, the numbers of injected PVs in each FTR are not the same

The ratios of the effluent organic compound concentrations to injected influent concentrations are presented in Fig. 3. The effluent concentrations from the Biocide FTR indicate the retardation of organic compounds due to the competitive sorption. While effluent benzene and toluene (Fig. 3a, b) concentrations from the Biocide FTR reached to ~ 90% of their injected value in less than 10 PVs, effluent naphthalene and 1-methylnaphthalene concentrations from the Biocide FTR (Fig. 3c, d) gradually reached to ~ 85% of their injected values after ~ 20 PVs and saturation of the soil adsorption capacity. The retardation of the injected organic compounds followed the order of benzene < toluene < naphthalene < 1-methylnaphthalene. This behavior suggested that PAH compounds have higher sorption capacity which can be attributed to their higher organic carbon-water partitioning coefficient (Ngueleu et al. 2018). The organic carbon-water partitioning coefficient (K_OC_) indicates the mobility and distribution of the organic compounds in the soil. A higher K_OC_ suggests more affinity to the organic matter of the soil and hence, can be a representative of larger sorption capacity (Jagiello et al. 2014). In addition, PAHs are generally more hydrophobic compared with BTEX which can also enhance PAH sorption capacity (Pignatello and Xing 1995). Once the PAHs were omitted from the injected solution (80 PVs), effluent benzene and toluene concentrations from the Biocide FTR decreased to 83 and 72% of the injected concentrations, respectively, because their concentrations in the injected synthetic solution were increased from 3.5 to 5 mg L^−1^ that resulted in a higher sorption capacity. Dissolved phase concentrations can significantly influence the sorption capacity based on different sorption isotherms (Weber and DiGiano 1995). Hence, increasing toluene and benzene concentrations enhanced their sorption capacity in the soil and consequently, their effluent concentrations decreased. After injecting ~ 10 PVs following the removal of PAH compounds, effluent benzene and toluene concentrations from the Biocide FTR again reached to ~ 90% of the injected values due to saturation of the sorption capacity until the end of the experiments.

The effluent benzene concentrations from the LS, BW, and CO FTRs fluctuated in the range of 85 to 90% during the initial 80 PVs (see Fig. 3a) and no significant difference was observed between the removal of benzene in the Biocide FTR and that in the LS, BW, HS, and CO FTRs. The effluent benzene concentrations from the HS FTR were slightly lower than those from the other FTRs which can be attributed to the higher adsorption capacity of the soil under higher salinity conditions (Wang et al. 2015; Ngueleu et al. 2018; Spaltro et al. 2019; Zhao et al. 2019). By increasing the salinity and ionic strength in the aqueous phase, solubility of organic compounds decreases which referred to as the salting-out effect (Endo et al. 2012) that influences the mass transfer process and results in a higher organic sorption in the soil. After omitting naphthalene and 1-methylnaphthalene from the injected solution (80 PVs), initially effluent benzene concentrations decreased for all FTRs due to higher equilibrium sorbed concentrations. After around 85 PVs, while effluent benzene concentrations from the CO FTR again increased to ~ 90% similar to those from the Biocide FTR, they appeared to decrease slightly for the LS, BW, and HS FTRs compared with those from the Biocide and CO FTRs. This behavior suggested that following the removal of PAH compounds and enhancement of the reducing conditions, benzene was slightly degraded under sulfate-reducing conditions. However, the results from the CO FTR indicate that while reducing condition was enhanced after the removal of PAHs, benzene was not affected under methanogenic conditions. This can be attributed to the slow growth of methanogens and because they require more strict anaerobic and reducing conditions (Gieg et al. 2014; Meckenstock et al. 2015). Sela-Adler et al. (2017) investigated the coexistence of SRB and methanogenic bacteria in an estuarine system under different sulfate concentrations and reported that methanogenesis rates were two orders of magnitude lower than those for sulfate reduction. Consequently, the relative contribution of methanogenic degradation in the benzene biodegradation process is not expected to be significant (Meckenstock et al. 2015).

Effluent toluene concentration from the CO FTR initially increased and fluctuated around 90% of the injected value similar to that from the Biocide FTR. After injecting ~ 50 PVs, effluent dissolved toluene from the CO FTR gradually decreased and reached to ~ 60% of the injected concentration by the end of the experiments. For the LS, BW, and HS FTRs, effluent toluene concentrations reached to a maximum concentration during the initial 30 PVs and then gradually decreased to ~ 40% of the injected value which is coincident by the enhancement of reducing conditions (Fig. 2) and can be attributed to the development of microbial community. For the HS FTRs, effluent toluene concentrations were similar to those of benzene and slightly lower than those from the LS and BW FTRs that can be attributed to the salting-out effect and higher adsorption capacity as the salinity and ionic strength increased (Endo et al. 2012); however, the biodegradation of toluene in the sulfate-rich environment was not significantly different for various salinity conditions. Toluene mass balance analyses indicate that at the beginning of injection, ~ 10% of the injected mass was degraded in the LS, BW, and HS FTRs and the remaining consumed mass was lost by adsorption. At the end of the experiments, ~ 50% of the injected toluene mass was degraded in the sulfate-rich FTRs while less than 25% of that in the CO FTR was degraded under potential methanogenic condition.

Effluent naphthalene concentrations gradually increased during the first 20 PVs and then fluctuated around 85% of the injected concentrations. After injecting 60 PVs, effluent naphthalene concentrations from the LS, BW, and HS FTRs appeared to decrease slightly relative to the CO and Biocide FTRs. Mass balance analyses indicated that ~ 5% of the injected naphthalene was degraded in the sulfate-rich conditions between the 60 and 80 PVs. As opposed to the behavior of benzene and toluene, effluent naphthalene concentrations from the HS FTRs were not lower than those from the LS and BW FTRs and thus, consistent with findings by Ngueleu et al. (2018) where they indicated that naphthalene sorption was not affected by changes in salinity for the same soil samples used in this study. This was attributed to the high affinity of naphthalene to organic carbon which provides a high sorption capacity and hence, salinity and salting-out effect cannot control the adsorption process. While for BTEX compounds, the lower affinity to organic carbon provides lower sorption capacity and hence, salting-out effect can dominate and enhance the adsorption process.

Effluent 1-methylnaphthalene concentrations from the LS, BW, and HS FTRs increased during the initial 20 PVs similar to the naphthalene behavior due to saturation of soil adsorption capacity and then gradually decreased and reached to ~ 65% of the injected concentration by the end of the injection period. Mass balance analyses indicated that ~ 5 and ~ 15% of the injected 1-methylnaphthalene mass were biodegraded at the beginning and end of the injection, respectively, for the LS, BW, and HS FTRs. For the CO FTR, effluent 1-methylnaphthalene concentrations were not significantly different between the CO and Biocide FTRs during the 70 PVs; however, during the last 10 PVs, around 5% of the injected 1-methylnaphthalene mass was utilized under potential methanogenic conditions.

Overall, the results from the LS, BW, and HS FTRs indicate that salinity did not affect treatment efficiency. In addition, degradation of injected organic compounds in the sulfate-rich conditions followed the order of toluene > 1-methylnaphthalene > naphthalene > benzene. While preliminary experiments with dissolved naphthalene as a single carbon source indicated that it could be utilized under sulfate-reducing conditions, in this study for the multi-component dissolved organic phase, naphthalene slightly degraded after injecting > 60 PVs. Benzene was degraded after removal of PAHs and enhancement of reducing conditions. Around 15% of the injected 1-methylnaphthalene mass was degraded under sulfate-reducing conditions and toluene was the only compound that was significantly degraded under both sulfate reduction and potential methanogenic conditions. Degradation under methanogenic conditions was lower than that under sulfate-reducing conditions which is consistent with the study by Sela-adler et al. (2017) who reported methanogenesis rates can be orders of magnitude lower than those from SRB. Other previous studies also indicated that toluene is one of the most biodegradable hydrocarbons under both sulfate-reducing (Edwards et al. 1992; Edwards and Garbic-Galic 1992) and methanogenic conditions (Edwards and Grbic-Galic 1994; Grbic-Galic and Vogel 1987) and usually it is the first organic compound utilized in the contaminated systems under different environmental conditions (Meckenstock et al. 2004). Because naphthalene was degraded in the preliminary LS FTRs with Eh values of + 300 mV, it is not expected that the biodegradation of benzene and PAHs is thermodynamically restricted because Eh values in this study reached to < 300 mV after 40 PVs. Meckenstock et al. (2016) proposed that the little energy conservation during anaerobic benzene or PAH degradation leads to extremely slow growth of bacteria that probably limits the kinetics of sulfate reduction rather than the thermodynamic aspects. Consequently, slight degradation of naphthalene can be attributed to the insufficient microbial populations which can degrade naphthalene. In addition, it can be hypothesized that the anaerobic degradation of toluene as a preferred carbon source could exert catabolic repression which restricts simultaneous utilization of other organic compounds. Meckenstock et al. (2004) also reported that toluene inhibited o-xylene degradation and following the removal of toluene from the system, o-xylene was degraded. They isolated two different types of SRB species from their soil samples and found that while one of the strains could degrade both toluene and o-xylene as the sole substrate, presence of toluene could inhibit o-xylene degradation. Moreover, since preliminary experiments with single dissolved naphthalene indicated salinity could affect the naphthalene utilization under sulfate-reducing conditions, it can be speculated that dominated microbes involved in toluene degradation are not susceptible to changes in salinity conditions under experimental conditions and toluene biodegradation dominated over salinity effects. Hence, similar reducing conditions (Fig. 2) and organic utilization (Fig. 3) were observed for different salinity conditions. It is concluded that the overall effect of salinity on multi-component dissolved organic phase degradation under sulfate-reducing conditions depends on the behavior of the specific target organic compounds that their degradation is dominant in the system.

### Sulfate reduction and DIC generation

**Fig. 4:**
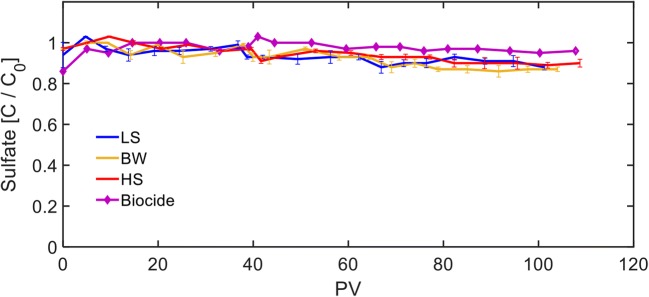
Ratio of effluent sulfate concentrations to the injected sulfate concentrations from the flow-through reactors (FTRs). Injected sulfate concentration was 150 mg L^−1^. Each data point for the low-salinity (LS), brackish water (BW), and high-salinity (HS) FTRs represents the average of single measurements from the duplicate reactors and the error bars represent the range of data. Each data point for the Biocide FTR is related to single measurement from one reactor

The ratios of the effluent sulfate concentrations to initial injected concentrations are shown in Fig. 4. As expected, the Biocide FTR had the highest effluent sulfate concentration during the experimental period. The effluent sulfate concentrations from the LS, BW, and HS FTRs initially increased to a maximum concentration of > 95% in less than 5 PVs. By the end of the experiments, the effluent sulfate concentrations gradually decreased and reached ~ 90% of the injected value that can be attributed to the enhancement of the reducing conditions (Fig. 2) and development of SRB-dominated community. No significant difference was observed between the sulfate consumptions under different salinity conditions. Overall, the sulfate consumption was ~ 10% of the injected mass because the injected sulfate concentration was very high to ensure SRB development over potentially competing microbes (Dar et al. 2008; Stams et al. 2005; Talbot et al. 2008). Toluene and 1-methylnaphthalene were the only organic compounds that were partially degraded under sulfate-reducing conditions and the injected sulfate concentrations were 9 and 20 times more than the theoretical value required to completely degrade the injected toluene and 1-methylnaphthalene, respectively, according to the following reactions (Cunningham et al. 2001; Meckenstock et al. 2016):

1 $$ {\mathrm{C}}_7{\mathrm{H}}_8+4.5\kern0.5em {{\mathrm{SO}}_4}^{2-}+3\ {\mathrm{H}}_2\mathrm{O}\to 2.25\ {\mathrm{H}\mathrm{S}}^{-}+2.25\ {\mathrm{H}}_2\mathrm{S}+7\ {{\mathrm{H}\mathrm{CO}}_3}^{-}+0.25\ {\mathrm{H}}^{+} $$

2 $$ {\mathrm{C}}_{11}{\mathrm{H}}_{10}+6.75\ {{\mathrm{SO}}_4}^{2-}+6\ {\mathrm{H}}_2\mathrm{O}\to 11\ {{\mathrm{H}\mathrm{CO}}_3}^{-}+6.75\ {\mathrm{H}\mathrm{S}}^{-}+4.25\ {\mathrm{H}}^{+} $$

**Fig. 5:**
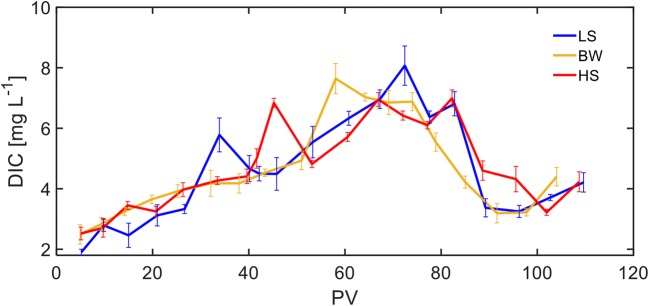
Effluent dissolved inorganic carbon (DIC) concentrations from the flow-through reactors (FTRs). Each data point represents the average of four measurements from the duplicate FTRs and the error bars represent the standard deviation

Complete mineralization of the total hydrocarbon mass is usually not expected that can be attributed to the thermodynamic limitations, limited mass transfer into the bacterial cell, and, furthermore, slow degradation rates due to recalcitrant molecular structures (Meckenstock et al. 2015). Effluent DIC concentrations as an indicator of complete mineralization of the injected organic compounds were measured in this study and the results are shown in Fig. 5. As expected, no significant difference was observed between the effluent DIC concentrations from the FTRs because the redox conditions as well as partial utilization of toluene and 1-methylnaphthalene were similar in all of them. DIC concentrations were initially around 2.5 mgL^−1^ and gradually increased to the range of 6–7 mg L^−1^ after 80 PVs. Following the removal of PAHs (80 PVs), effluent DIC concentrations decreased to around 3 mg L^−1^ but gradually increased to > 4 mg L^−1^ by the end of the experiments. The decrease of DIC concentrations can be attributed to the removal of 1-methylnaphthalene as a carbon source as well as the disturbance of SRB community. Assuming that all of the DIC concentrations at the end of experiments are related to the toluene mineralization, while ~ 60% of the injected toluene mass was lost due to adsorption and biodegradation, just around 20% of that was completely degraded by SRB according to Reaction 1.

## Summary and conclusions

An experimental flow-through system representing anaerobic utilization of petroleum hydrocarbons under sulfate-reducing conditions was developed to investigate the effects of salinity and dissolved organic phase composition under realistic dynamic subsurface conditions. The results indicated that retardation and adsorption capacity of soil for PAHs were more than those for BTEX that was attributed to the higher organic carbon-water partitioning coefficient. Benzene and toluene had higher adsorption capacity at higher salinity conditions due to salting-out effect. PAH sorption was not affected by changes in salinity due to high affinity of naphthalene to organic carbon which provides a high sorption capacity and hence, salinity and salting-out effect cannot control the adsorption process. Degradation of injected organic compounds in the sulfate-rich conditions followed the order of toluene >1-methylnaphthalene > naphthalene > benzene. While salinity did not affect treatment efficiency, it was indicated that substrate interactions and dissolved phase composition can result in rate-limited and insufficient biodegradation. It was indicated that dominant toluene degradation as a preferred carbon source in this study exerts catabolic repression on the simultaneous utilization of other organic compounds. Monitoring of the geochemical indicators suggested that microbes involved in toluene degradation were not susceptible to changes in salinity conditions, and hence, similar reducing conditions and organic utilization were observed for different salinity conditions. It can be concluded that the overall effect of salinity on multi-component dissolved organic phase degradation under sulfate-reducing conditions depends on the behavior of the target organic compounds that their degradation is dominant in the system. The assembled data set from this study provides a unique and comprehensive insight into the organic compound interactions under sulfate-reducing and different salinity conditions within a realistic dynamic anaerobic system.

## Electronic supplementary material

ESM 1 (PDF 122 kb)
