# Supplementary material for: Effects of dissolved organic phase composition and salinity on the engineered sulfate application in a flow-through system
Source: Environ Sci Pollut Res Int. 2020 Jan 24;27(11):11842–54. doi: 10.1007/s11356-020-07696-6 (PMC7136190; doi:10.1007/s11356-020-07696-6)
Supplement: Supplementary file 1 — (PDF 122 kb) [file 11356_2020_7696_MOESM1_ESM.pdf]

Effects of dissolved organic phase composition and salinity on the engineered sulfate application in a flow-through system

Supplementary Materials

Journal of Environmental Science and Pollution Research

Saeid Shafieiyoun[1], Riyadh I. Al-Raoush[1,*], Reem Elfatih Ismail[1], Stephane K. Ngueleu[1,2], Fereidoun Rezanezhad[2], and Philippe Van Cappellen[2]

[1]Department of Civil and Architectural Engineering
College of Engineering, Qatar University, PO Box 2713
Doha, Qatar

[2]Ecohydrology Research Group and Water Institute,
Department of Earth and Environmental Sciences,
University of Waterloo, 200 University Avenue West,
Waterloo, Ontario, Canada N2L 3G1

*corresponding author; e-mail: riyadh@qu.edu.qa

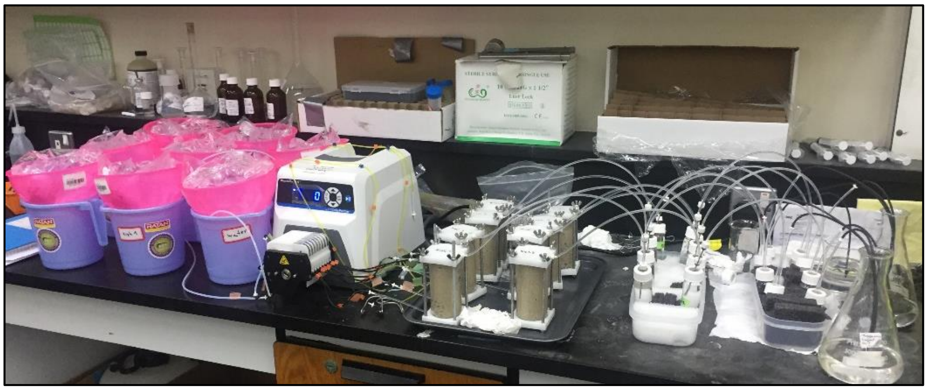

Figure SM1: Photo of the flow-through reactor (FTR) setup

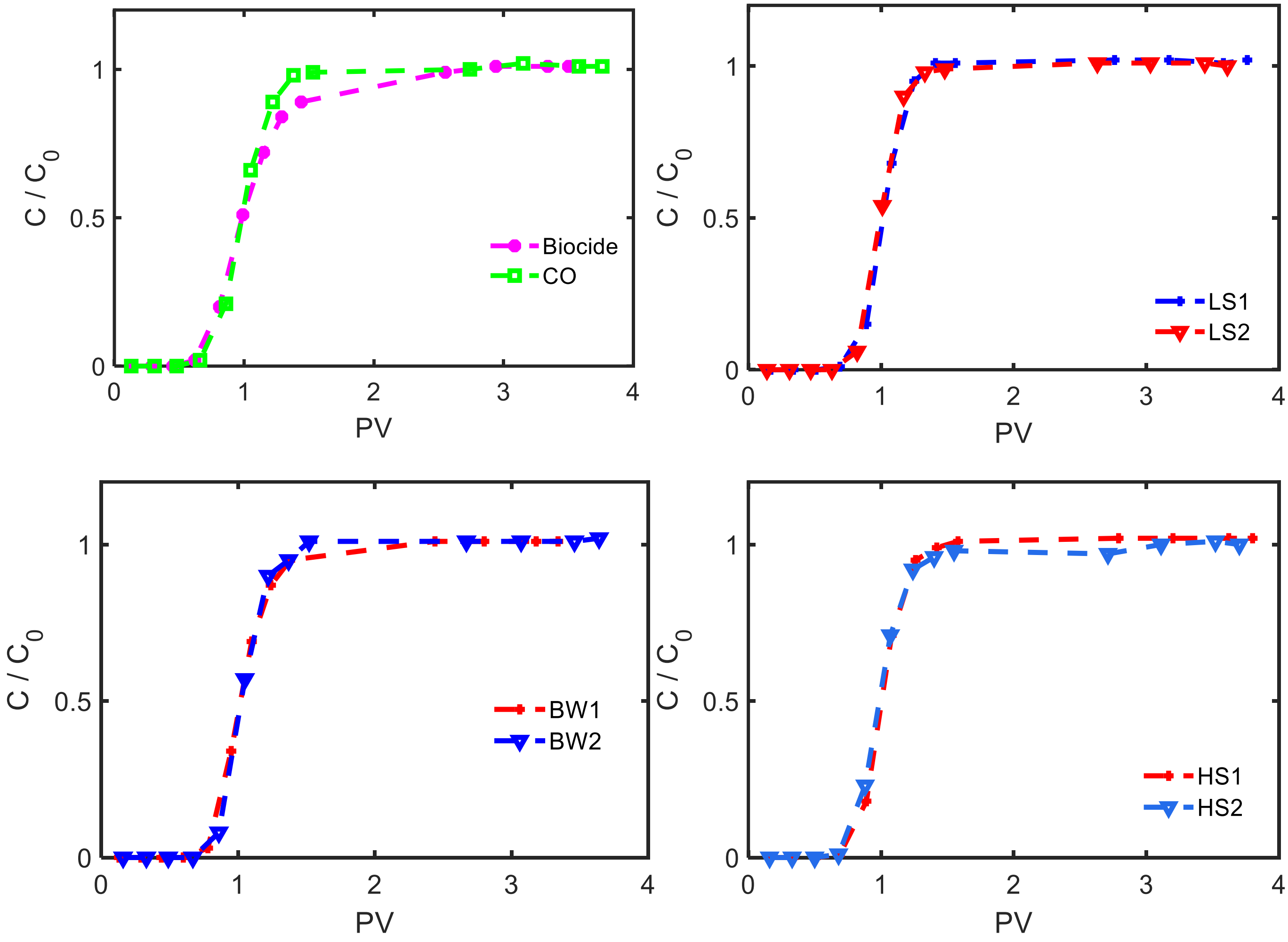

Figure SM2: Tracer test results for a) Biocide and CO FTRs, b) low salinity (LS1 and LS2) FTRs, c) brackish water (BW1 and BW2) FTRs, d) high salinity (HS1 and HS2) FTRS.
